# Frequency of Restless Legs Syndrome in Pakistani Patients With Type 2 Diabetes Mellitus

**DOI:** 10.7759/cureus.62624

**Published:** 2024-06-18

**Authors:** Nauman Ismat Butt, Muhammad Sohail Ajmal Ghoauri, Umaima Waris, Dure Sabeh, Fahad Qaisar

**Affiliations:** 1 Internal Medicine/Rheumatology, Azra Naheed Medical College, Superior University, Lahore, PAK; 2 Medicine/Neurology, Bahawal Victoria Hospital, Quaid-e-Azam Medical College, Bahawalpur, PAK; 3 Medicine, Azra Naheed Medical College, Superior University, Lahore, PAK; 4 Medicine, Bahawal Victoria Hospital, Quaid-e-Azam Medical College, Bahawalpur, PAK

**Keywords:** diabetic nephropathy (dn), diabetic retinopathy, diabetic peripheral neuropathy (dpn), diabetes mellitus type 2, restless leg syndrome (rls)

## Abstract

Objective

To determine the frequency of restless legs syndrome (RLS) among Pakistani patients with type 2 diabetes mellitus.

Methods

This observational cross-sectional study was carried out in the Department of Medicine at Bahawal Victoria Hospital, Quaid-e-Azam Medical College, Bahawalpur, Pakistan, from January 2024 to May 2024. The National Institute of Health (NIH) diagnostic criteria were used to diagnose RLS. Type 2 diabetes mellitus was defined as patients with an HbA1c greater than 7.0%, two random blood glucose readings of ≥200 mg/dL, a previous history of diabetes diagnosis, or those taking anti-hyperglycemic medicines. Patients with a history of leg surgery or amputation, iron deficiency anemia, alcoholism, end-stage kidney disease, chronic liver disease, those on hemodialysis, and pregnant women were excluded from the study. After ethical approval and informed consent were obtained, 255 patients with type 2 diabetes mellitus were included in the study using a non-probability consecutive sampling technique. Demographic information including age, gender, and duration of diabetes was noted, and patients were assessed for diabetes control, peripheral neuropathy, retinopathy, and RLS Patient records were assessed for HbA1c levels and urine examination to diagnose nephropathy. All data were entered into SPSS version 23. A Chi-Square test was applied post-stratification using a p-value of less than 0.05 as significant.

Results

The mean age was 53.5 ± 12.8 years with 140 (54.9%) females. The mean duration of the disease and mean HbA1c were 6.8 ± 5.4 years and 9.8 ± 2.5%, respectively, with 191 (74.9%) patients having poor control of diabetes. Peripheral neuropathy was seen in 131 (51.4%) patients, retinopathy in 58 (22.7%), and nephropathy in 23 (9.0%). RLS was present in 34 (13.3%) patients with type 2 diabetes mellitus, showing a significant association with diabetes control (p-value = 0.001), peripheral neuropathy (p-value = 0.016), retinopathy (p-value = 0.006), and nephropathy (p-value = 0.011), but not with age (p-value = 0.122), gender (p-value = 0.217), or duration of diabetes (p-value = 0.922).

Conclusion

RLS was not an uncommon finding in patients with type 2 diabetes mellitus, being more common among those with poor diabetes control and the presence of other complications such as neuropathy, nephropathy, and retinopathy.

## Introduction

Restless legs syndrome (RLS) is clinically manifested by an urge for limb movement along with an unpleasant sensation that is relieved by moving the limbs. Symptoms typically worsen in the evening and nocturnal hours, often waking the patient from sleep [1]. The dopamine neurons of the substantia nigra, which play a role in movement modulation, may dysfunction due to iron deficiency in these neurons, contributing to the pathophysiology of RLS. Unlike in the brain, iron levels may usually be normal elsewhere in the body. The prevalence of RLS varies from 2% to 12% in the adult population in the Western world [1], but in Asian countries, it has been reported to be as low as 0.1% [2]. RLS can be primary or secondary to disorders such as iron deficiency anemia, chronic kidney disease, pregnancy, autoimmune rheumatic diseases, Parkinson’s disease, and multiple sclerosis [3]. There are no specific diagnostic tests for RLS; diagnosis is usually made clinically using the National Institutes of Health (NIH) diagnostic criteria [4].

Recent studies have indicated that RLS is significantly more common in patients with diabetes mellitus than in the general population, affecting one in four diabetic patients [5]. Merlino G et al. reported that RLS was present in 17.7% of diabetic patients in Italy [6], while Mirghani H found RLS in 31.7% of Sudanese patients with type 2 diabetes mellitus [1]. Studies on the association of RLS with diabetes mellitus from Pakistan are scant. A study from Islamabad by Safdar et al. reported no association between RLS and diabetes mellitus [7], whereas Nawaz MS et al. reported RLS in 81.7% of diabetics [8]. Siddiqi SA et al. observed RLS in 55.8% of patients with diabetes mellitus [9]. Other studies on the Pakistani population have investigated its association with obesity, pregnancy, and renal failure. Malik LA et al. reported RLS in 43.2% of obese individuals, associating it with increasing age, physical inactivity, and insomnia [10]. Siddique S et al. demonstrated RLS in 38.7% of patients with end-stage renal disease in Lahore, Pakistan, with no association with age, gender, disease duration, smoking, or alcohol use [11]. In pregnant females in Lahore, Pakistan, RLS was seen in 25.4% of patients [12].

Diabetes mellitus, a global health burden, currently affects 285 million people [13]. It is projected that 438 million people will have diabetes by the year 2030, with Asian populations bearing the major bulk of the disease burden [13]. Despite improvements in management, the incidence of microvascular complications and comorbidities of diabetes, including neuropathy, nephropathy, and retinopathy, remain high, causing significant morbidity, with cardiovascular disease being the leading cause of death [1,14]. There is a great variation in the global prevalence of RLS among diabetic patients. Furthermore, there is a severe paucity of data regarding these two conditions in the Pakistani population. Due to ethnic differences, healthcare resources, and disease awareness, studies conducted in other countries may not be applicable to Pakistan. Therefore, we conducted the present study to determine the frequency of RLS in Pakistani patients with type 2 diabetes mellitus.

## Materials and methods

The present observational cross-sectional study was carried out at the Department of Medicine, Bahawal Victoria Hospital, Quaid-e-Azam Medical College, Bahawalpur, Pakistan, from January 2024 to May 2024, to determine the frequency of RLS among patients with type 2 diabetes mellitus. The NIH diagnostic criteria were used to define RLS as 'an urge to move the legs, usually accompanied or caused by uncomfortable or unpleasant sensations in the legs; beginning or worsening during periods of rest or inactivity such as lying or sitting; partially or totally relieved by movement such as walking or stretching; worse in the evening or at night than during the day, or only occurring in the evening or night' [4]. Type 2 diabetes mellitus was defined as patients with an HbA1c greater than 7.0%, two random blood glucose readings of ≥200 mg/dl, a previous history of diabetes diagnosis, or those taking anti-hyperglycemic medicines [15]. Nephropathy was identified by the presence of microalbuminuria on urinalysis, a urine albumin-creatinine ratio of 30-300 mg/g, and an eGFR of more than 60 ml/min per 1.73 m^2^ (indicative of early nephropathy without evidence of end-stage/chronic renal disease) [16]. Retinopathy was identified by the presence of microaneurysms, hard exudates, macular edema, and new vessel formation on ocular examination by an ophthalmologist [17]. Peripheral neuropathy was clinically identified by pain, paresthesia, and sensory loss in a glove-and-stocking pattern on the limbs at the time of examination [18]. Good diabetes control was defined as an HbA1c of less than or equal to 7.0%, whereas poor diabetes control was defined as an HbA1c greater than 7.0%.

Keeping a 95% CI with a 5% margin of error, a sample size of 224 was calculated based on an expected frequency of RLS of 17.7% in patients with type 2 diabetes mellitus [6]. Excluded from the study were patients with peripheral vascular disease, those with a history of leg surgery or amputation, those with iron deficiency anemia, alcoholism, autonomic neuropathy, end-stage kidney disease, chronic liver disease, those on hemodialysis, those with neurological diseases such as Parkinson's Disease, those on antidepressant therapy, and pregnant women. After ethical approval and obtaining informed consent, 255 patients with type 2 diabetes mellitus, as per the operational definition, were included in the study using a non-probability consecutive sampling technique. Demographic information including age, gender, and duration of diabetes was noted, and patients were clinically assessed for diabetes control, peripheral neuropathy, retinopathy, and restless legs syndrome. Patient records were assessed for HbA1c levels and urine examination to identify nephropathy. All data were recorded and entered into SPSS version 23 for analysis. Means and SDs were calculated for numerical data, whereas percentages and frequencies were generated for qualitative variables. A Chi-square test was applied post-stratification using a p-value of less than 0.05 as significant.

## Results

A total of 255 patients with type 2 diabetes mellitus were enrolled in the present study, having a mean age of 53.5±12.8 years, with 129 (50.6%) aged 54 years or older. The majority of the patients were female (n=140, 54.9%), as shown in Table 1. The mean duration of the disease was 6.8±5.4 years, with 137 (53.7%) having a disease duration of 6 years or more. Twenty-five patients (9.8%) were newly diagnosed with diabetes mellitus at this presentation. The mean HbA1c was 9.8±2.5%, and 191 (74.9%) had poor control of their diabetes, as shown in Table 1. With regards to complications of diabetes mellitus, peripheral neuropathy was seen in 131 (51.4%) patients, retinopathy in 58 (22.7%), and nephropathy in 23 (9.0%). In the present study, RLS was present in 34 (13.3%) patients with type 2 diabetes mellitus. Stratification of data demonstrated a significant statistical association of RLS with poor diabetes control (p-value = 0.001), presence of peripheral neuropathy (p-value = 0.016), presence of retinopathy (p-value = 0.006), and presence of nephropathy (p-value = 0.011) but not with age (p-value = 0.122), gender (p-value = 0.217), and duration of diabetes mellitus (p-value = 0.922), as shown in Table 2.

**Table 1:** Demographic and clinical variables of the diabetic patients.

Variables		Frequency (n)	Percent (%)
Gender	Female	140	54.9
	Male	115	45.1
Age	<53 years	126	49.4
	>54 years	129	50.6
Duration of Diabetes	<5 years	118	46.3
	>6 years	137	53.7
Diabetes Control	Good / Controlled	64	25.1
	Poor / Uncontrolled	191	74.9
Peripheral Neuropathy	Present	131	51.4
	Absent	124	48.6
Retinopathy	Present	58	22.7
	Absent	197	77.3
Nephropathy	Present	23	9.0
	Absent	232	91.0
Restless Legs Syndrome	Absent	221	86.7
	Present	34	13.3

**Table 2:** Stratification of demographic and clinical data with regards to restless legs syndrome.

Clinical Variables	Restless Legs Syndrome		P-value
	Absent	Present	
Gender:			0.217
Female	118 (84.3%)	22 (15.7%)	
Male	103 (89.6%)	12 (10.4%)	
Age:			0.122
<53 years	105 (83.3%)	21 (16.7%)	
>54 years	116 (89.9%)	13 (10.1%)	
Duration of Diabetes:			0.922
<5 years	102 (86.4%)	16 (13.6%)	
>6 years	119 (86.9%)	18 (13.1%)	
Diabetes Control:			0.001
Good / Controlled	63 (98.4%)	01 (1.6%)	
Poor / Uncontrolled	158 (82.7%)	33 (17.3%)	
Peripheral Neuropathy:			0.016
Present	107 (81.7%)	24 (18.3%)	
Absent	114 (91.9%)	10 (8.1%)	
Nephropathy:			0.011
Present	16 (69.6%)	07 (30.4%)	
Absent	205 (88.4%)	27 (11.6%)	
Retinopathy:			0.006
Present	44 (75.9%)	14 (24.1%)	
Absent	177 (89.8%)	20 (10.2%)	

## Discussion

There are numerous studies documenting the frequency of RLS in patients with type 2 diabetes mellitus, but this association is not solely explainable by the presence of peripheral neuropathy [1]. It has been postulated that prolonged sleep loss may result in insulin resistance and metabolic disturbances [19]. RLS was present in 13.3% of diabetic patients in this study. This is similar to the frequency reported by Merlino G et al., where 17.7% of diabetic patients in Italy were found to have RLS associated with neuropathy [6]. Mirghani H found RLS in 31.7% of Sudanese patients with type 2 diabetes mellitus [1], where it was associated with neuropathy but not with age, gender, retinopathy, or HbA1c [1]. In this study, a significant statistical association of RLS was observed with poor diabetes control (p-value = 0.001), neuropathy (p-value = 0.016), retinopathy (p-value = 0.006), and nephropathy (p-value = 0.011). Of the 64 patients with good control of diabetes mellitus, only 1 (1.6%) had RLS, compared to 33 (17.3%) of the 191 patients with poor diabetes control. Among the 131 patients with neuropathy, 24 (18.3%) had RLS, compared to 10 (8.1%) of the 124 without neuropathy. Of the 58 patients with retinopathy, 14 (24.1%) had RLS, whereas among the 197 patients without retinopathy, 20 (10.2%) reported RLS. Among the 23 patients with nephropathy, 7 (30.4%) complained of RLS, compared to 27 (11.6%) of the 232 without nephropathy. No statistical association of RLS was observed with age (p-value = 0.122), gender (p-value = 0.217), or duration of diabetes mellitus (p-value = 0.922). Regarding the Pakistani population, Nawaz MS et al. reported RLS to be present in 81.7% of diabetics, which is much higher compared to our study [8], and associated with age, gender, HbA1c, and education level [8]. Siddiqi SA et al. saw RLS in 55.8% of patients with diabetes mellitus [9], whereas Safdar M et al. reported no association between RLS and diabetes mellitus [7]. Sabic A et al. also demonstrated no association between RLS and type 2 diabetes mellitus [20]. This variance in RLS prevalence may be due to differences in study design, the presence of comorbidities, and environmental, ethnic, and behavioral factors [1,5,21].

Ning et al. reported RLS to be present in 25% of patients with diabetes mellitus, much higher than in the general population [5]. Furthermore, the prevalence of RLS was higher in Asian countries than non-Asian countries, with males affected almost twice as much as women [5]. Given the significant rise in diabetes in the Asian population, it is important to screen patients for comorbidities and complications of diabetes mellitus to reduce disease burden. Additionally, reduced insulin sensitivity has been reported in Asians compared to non-Asians, potentially increasing the likelihood of poor diabetes control and complication risk [22]. In animal models, RLS has been linked to the presence of various genes such as BTBD9 and MEIS1 involved in iron and ferritin regulation pathways [23]. However, the role of genetic differences in humans remains poorly investigated. The treatment of RLS requires evaluation to identify any secondary causes and triggering factors, which may then be treated and eliminated [3]. In cases of primary RLS, symptom management is the mainstay of treatment [24]. For patients with mild symptoms, educating patients about sleep hygiene and non-pharmacologic measures such as massage, cognitive distraction, walking, stretching, and cool/warm temperature baths are recommended [25,26]. However, for patients with more severe symptoms, pharmacologic therapy is warranted, including GABA analogues (gabapentin, pregabalin), dopamine agonists (pramipexole, ropinirole, rotigotine), or opioids (buprenorphine), depending on the associated comorbidities, pain, and motor symptoms [24,27].

The present study has certain limitations that should be considered as well. Based in a single hospital, the results of our study may not be generalizable to the broader population. It is therefore pertinent to carry out further studies with a larger sample size to highlight the role and importance of RLS in the Pakistani diabetic population. RLS is a potentially treatable condition associated with diabetes mellitus, especially neuropathy, and can negatively affect life and sleep quality. By raising awareness regarding RLS screening among patients and physicians, early diagnosis and prompt treatment could lead to a reduction in disease morbidity and improvement in the overall quality of life for these patients.

## Conclusions

RLS was seen in 13.3% of the 255 patients with type 2 diabetes mellitus enrolled in our study. A significant association of RLS was observed with diabetes control and the presence of other complications (neuropathy, retinopathy, nephropathy) but not with patient age, gender, or duration of diabetes. Therefore, we recommend optimization of diabetes control to help reduce the burden of complications and morbidities associated with type 2 diabetes mellitus.
